# Supplementary material for: Structures of Native Doublet Microtubules from Trichomonas vaginalis Reveal Parasite-Specific Proteins
Source: Nat Commun. 2025 Apr 29;16:3996. doi: 10.1038/s41467-025-59369-y (PMC12041511; doi:10.1038/s41467-025-59369-y)
Supplement: Supplementary file 1 — Supplementary Information [file 41467_2025_59369_MOESM1_ESM.pdf]

Supplementary Figures

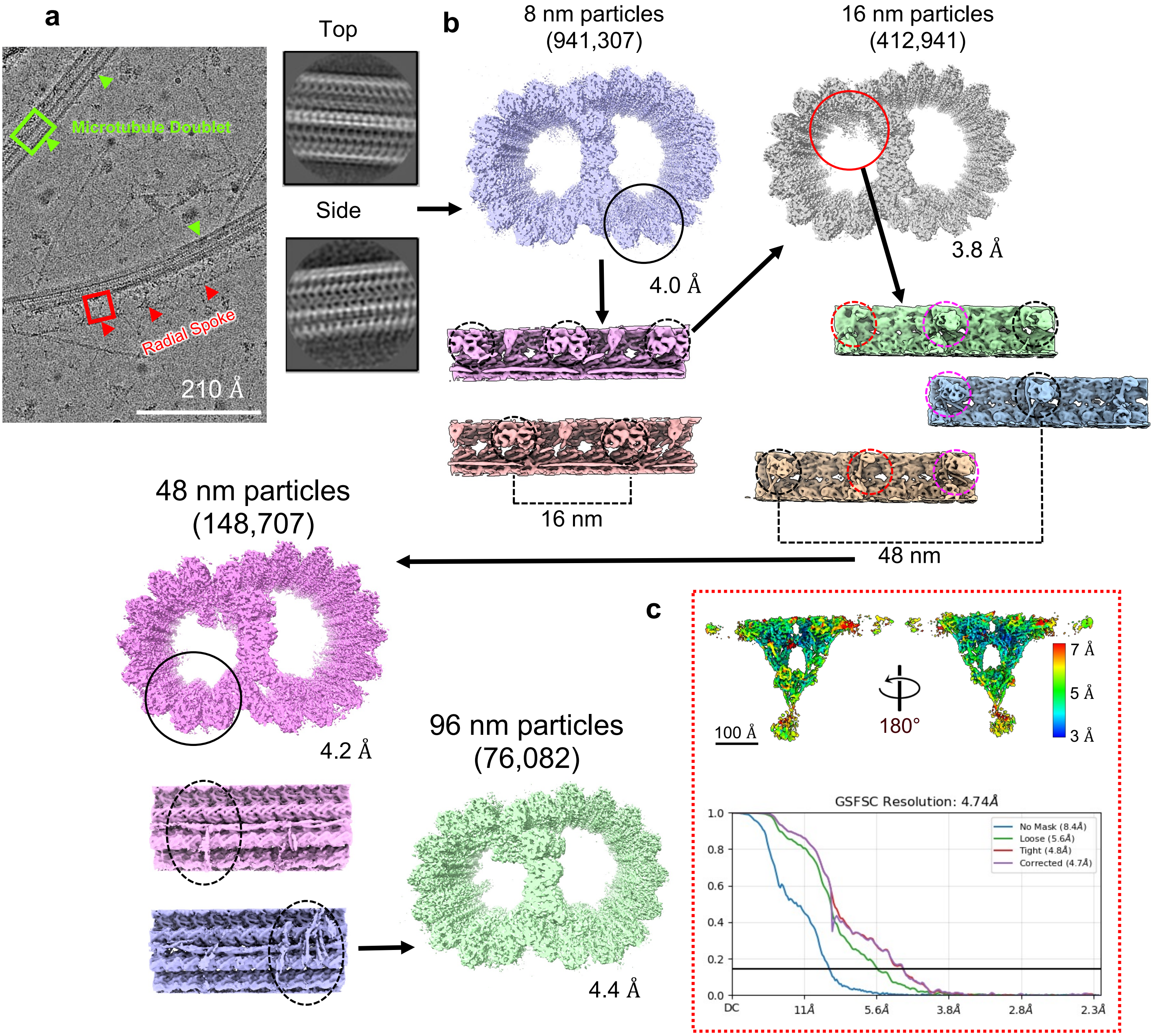

Supplementary Figure 1. Data Processing of *Tv*-DMTs. (**a**) Example cryoEM micrograph (low-pass filtered to 5 Å) with doublet microtubules (green arrowheads) and attached radial spokes (red arrowheads) and representative 2DClasses of microtubule doublets. (**b**) DMT particle processing pipeline. (**c**) Asymmetric reconstruction of *Tv* RS head colored by local resolution.

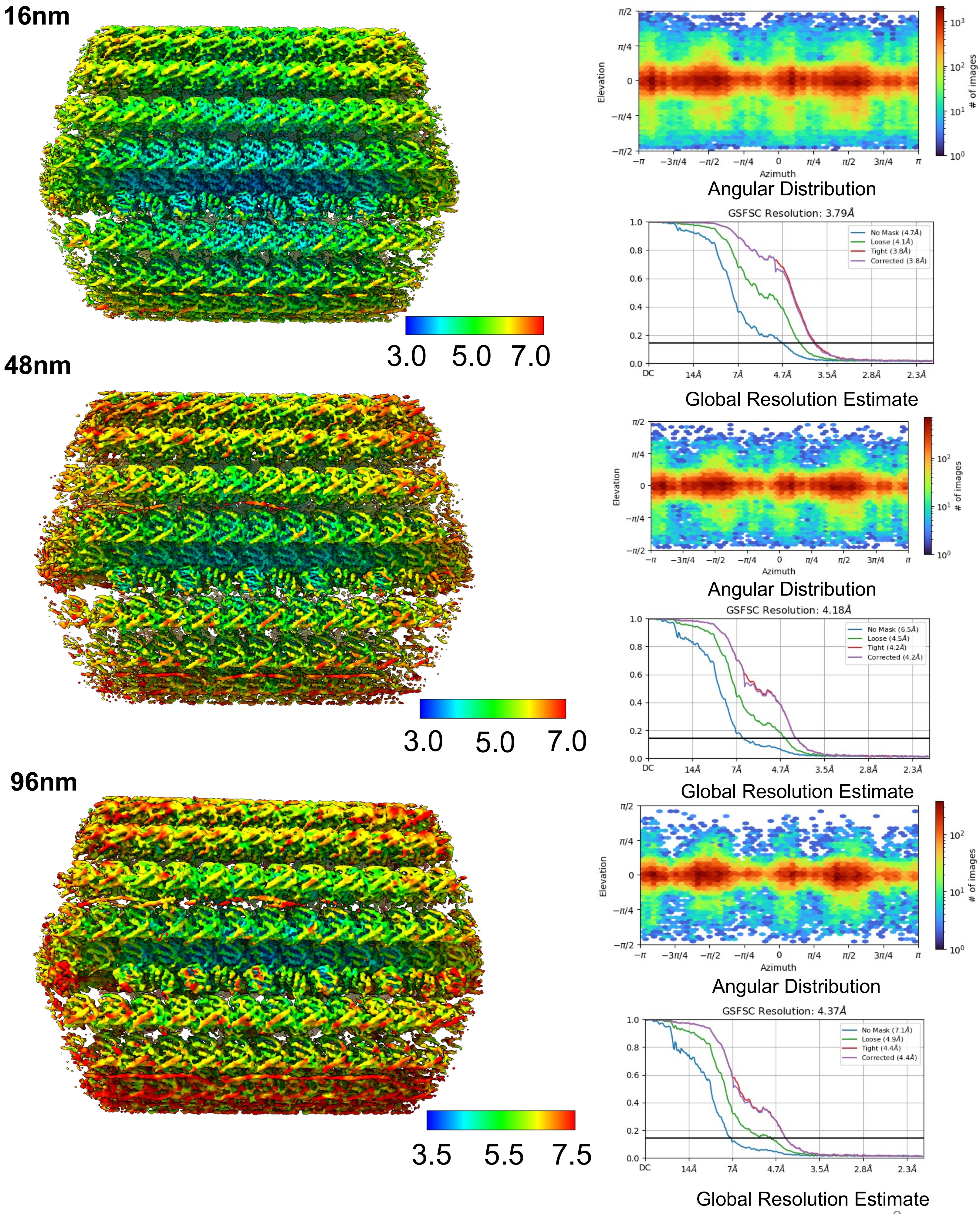

Supplementary Figure 2. Local Resolution, Angular Distribution of Particles, and Global Resolution Estimate with FSC Cutoff for 16, 48, and 96nm repeating *Tv*-DMT Maps

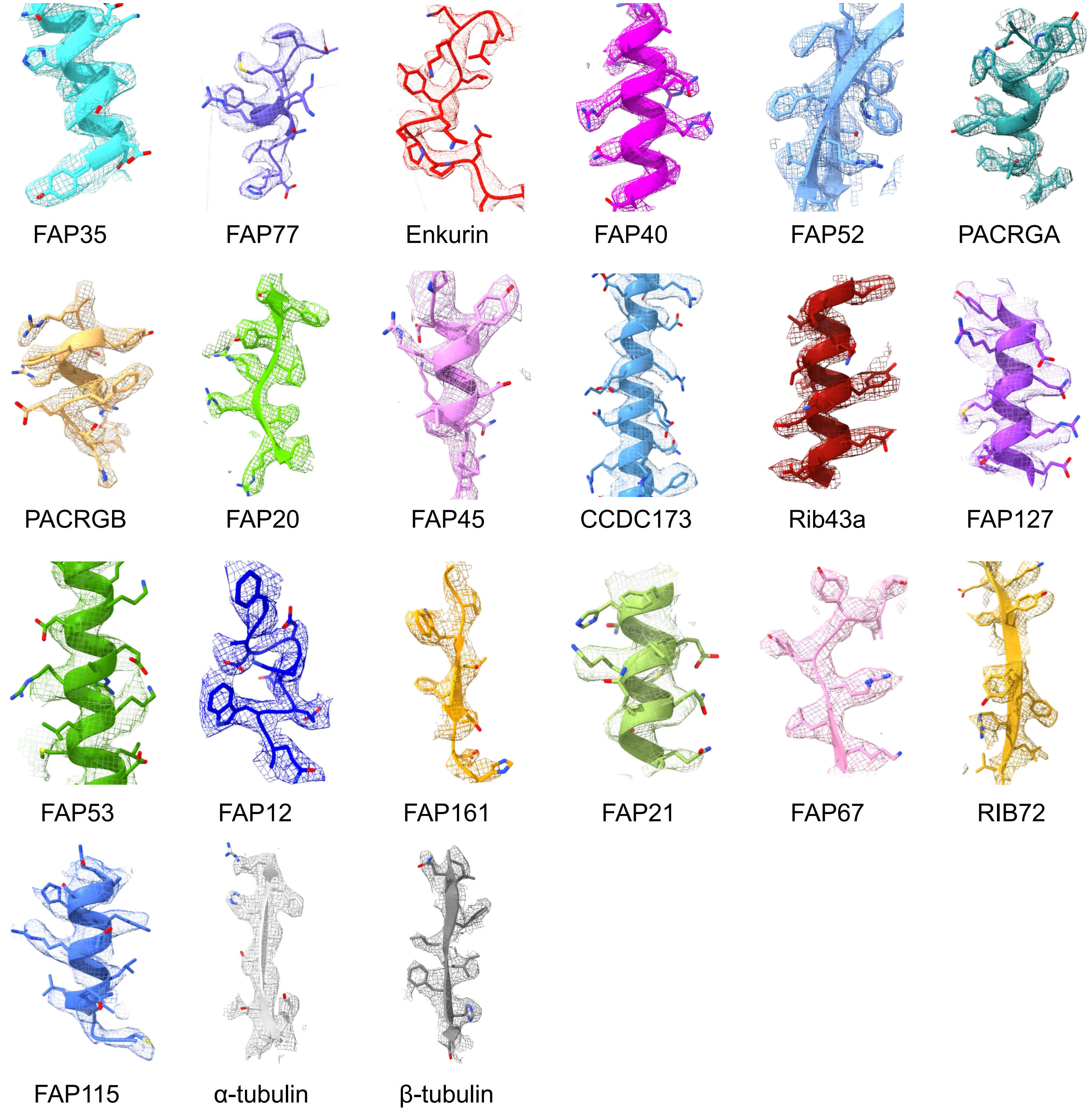

Supplementary Figure 3. Fitted models in cryoEM densities. Examples of cryoEM density maps (wireframes) with fitted atomic models (ribbons and sticks) of MIP and tubulin proteins.

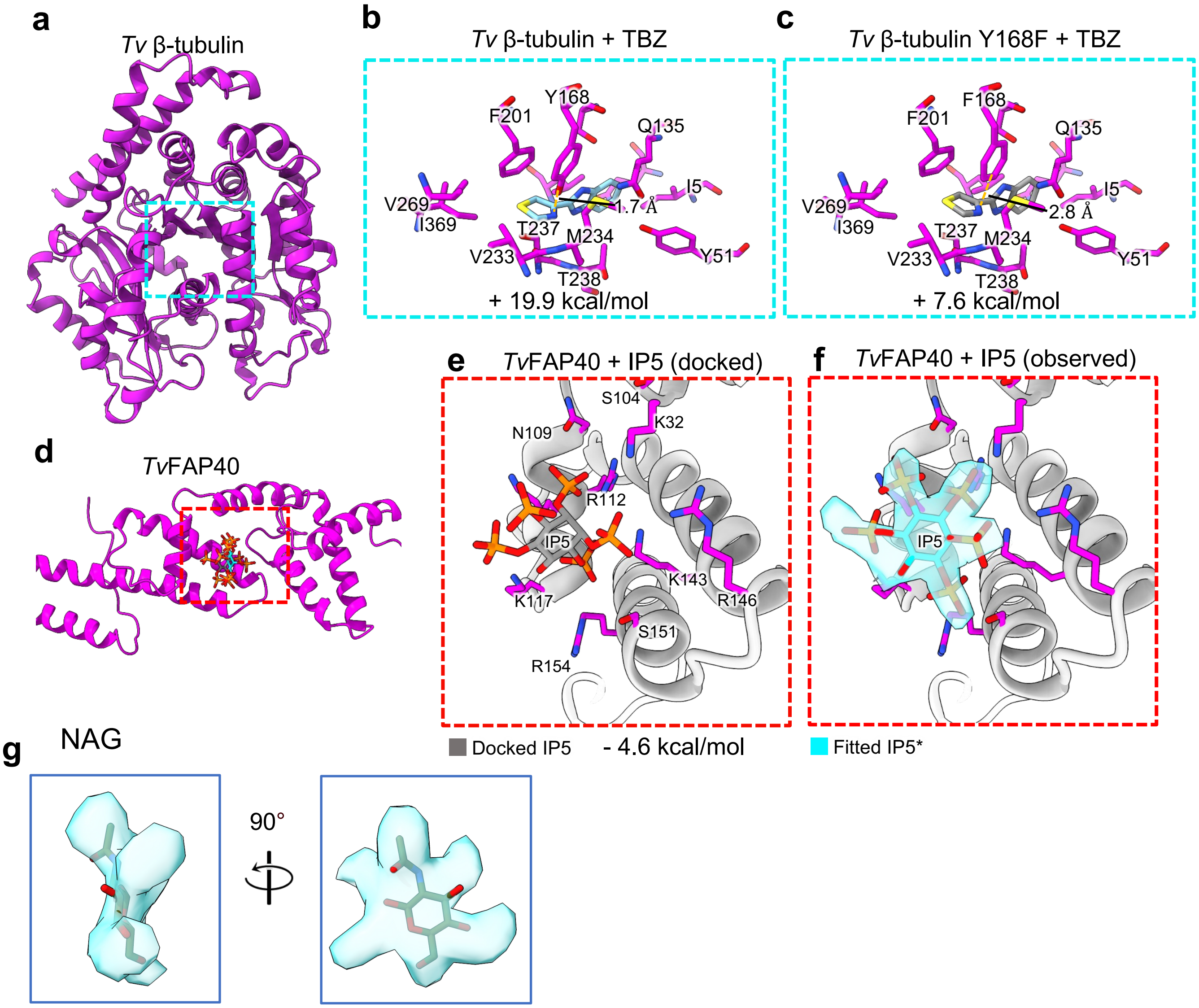

Supplementary Figure 4. Docking of β-tubulin and TvFAP40. (a) Atomic model of β-tubulin with putative BZ drug binding site boxed. (b) WT Tv β-tubulin with docked thiabendazole (TBZ), fit into putative binding site. (c) Tv β-tubulin Y168F mutant with docked TBZ in putative binding site. (d) Atomic model of TvFAP40 with putative binding site boxed. (e, f) TvFAP40 binding pocket with docked IP5 (yellow and red sticks) (e) and fitting into the cryoEM map density (semitransparent surface) (f). Docking of NAG into TV ligand spot (g)

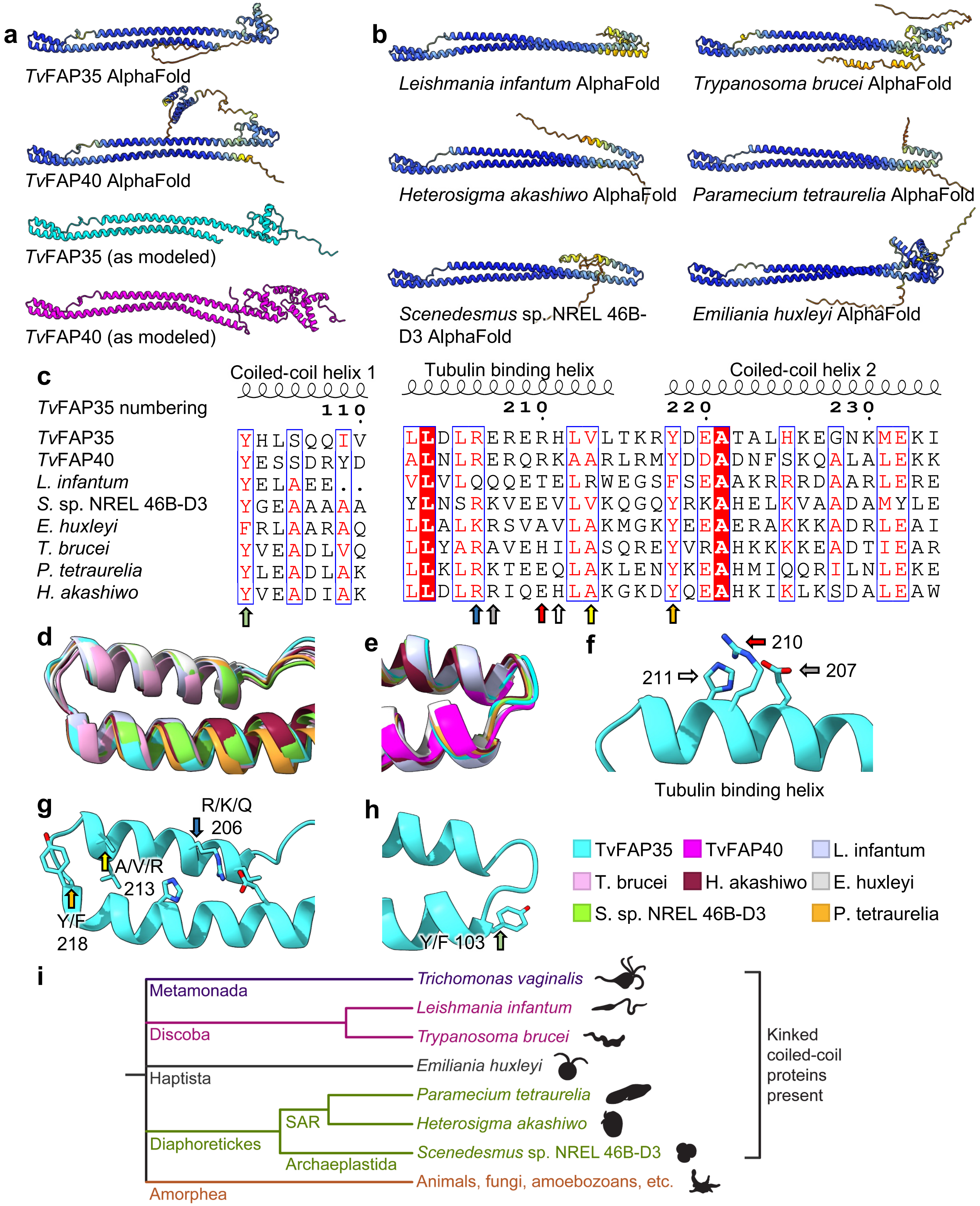

Supplementary Figure 5. Analysis of TvFAP40 and TvFAP35 and structural homologs. (a) AlphaFold-predicted models of TvFAP35 and TvFAP40 (top) colored by AlphaFold confidence interval (blue more confident, red less confident) and their atomic models (bottom) colored in cyan and magenta respectively. (b) AlphaFold-predicted structures for structural homologs from selected species, colored by AlphaFold confidence interval. (c) Sequence alignment of dimerization and MT binding domain regions from proteins in a and b aligned to TvFAP35, with conserved residues highlighted and those at the active site indicated with arrows. (d and e) α-carbon backbone aligned models from the MT-binding and dimerization domains of the kinked-coiled-coil domains. (f) Conserved proteins from c shown at their locations at the MT-binding interface on TvFAP35. (g-h) Same as f but based on both faces of the dimerization domain. (i) Phylogeny tree including organisms in which FoldSeek identified similar protein structures.

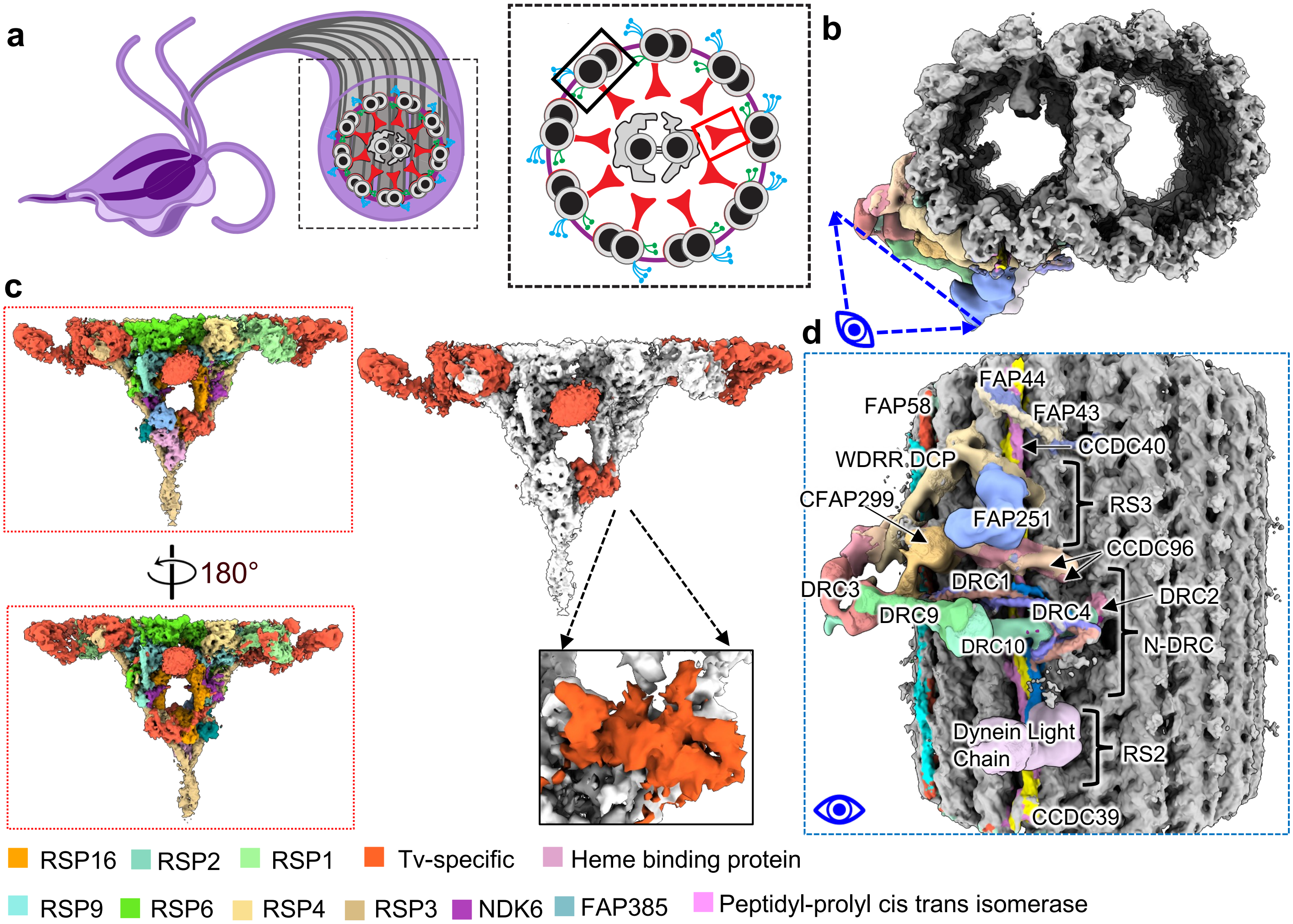

Supplementary Figure 6. Low Resolution structures of Tv-DMT associated axonemal components. (a) Diagram of Tv axoneme. (b) Cross sectional view of Tv-DMT with low resolution N-DRC related components labeled with various colors. (c) Low resolution reconstructions of Tv radial spokes with domains conserved in C. reinhardtii labeled in various colors (left) and Tv-specific domains are colored in red (left and right). (d) Side view of N-DRC-related proteins from b with proteins labeled based on C. reinhardtii homologs.

Supplementary Tables

Supplementary Table 1 CryoEM data collection

	WT *Tv*-DMT 16 nm repeat (EMD-46642)	WT *Tv*-DMT 48 nm repeat (EMD-46643)	WT *Tv*-DMT 96 nm repeat (EMD-46636)	WT *Tv*-DMT 48 nm composite (EMD-46580) (PDB 9D5N)	Radial spoke head (EMD-48446)
**Data collection and processing**					
Magnification	81,000	81,000	81,000	81,000	81,000
Voltage (kV)	300	300	300	300	300
Electron exposure (e–/Å^2)	45	45	45	45	45
Defocus range (μm)	-1.5 to -2.5	-1.5 to -2.5	-1.5 to -2.5	-1.5 to -2.5	-1.5 to -2.5
Pixel size (Å)	1.1	1.1	1.1	1.1	1.1
Symmetry imposed	C1	C1	C1	C1	C1
particle images (no.)	425,317	148,707	76,082	425,317	131,906
Map resolution (Å)	3.8	4.2	4.4		4.7
FSC threshold	0.143	0.143	0.143		0.143
Repeat unit (nm)	16	48	96	48	
Symmetry	C1	C1	C1	C1	C1
**Refinement**					
Initial model used (PDB code)	6u42				
Model resolution (Å)	4.2				

FSC threshold	0.143
Model resolution range (Å)	2.2–4.3
Map sharpening B factor (Å²)	N/A
**Model composition**	
Non-hydrogen atoms	1,449,702
Protein residues	182,234
Ligands	MG:188<br>GTP:188
**B factors (Å²)**	
Protein	0.00 / 851.46 / 121.60
Ligand	8.48 / 230.06 / 101.35
**R.m.s. deviations**	
Bond lengths (Å)	0.004
Bond angles (°)	0.985
**Validation**	
MolProbity score	1.54
Clashscore	6.94
Poor rotamers (%)	0.29
**Ramachandran plot**	

Favored (%)	97.09
Allowed (%)	2.90
Disallowed (%)	0.02

Supplementary Table 2 MIPS and MOPS: Conserved and Novel MIPs in *Tv*, * indicates identified proteins unmodeled in *Tv* microtubule .pdb file, contact author for AlphaFold [1] fit of these unmodeled proteins, Red text indicates Tv-specific proteins

	Location	Protein	Uniprot ID	Copy number in 96 nm repeat	Length (residues)	Modeled residues	*C. reinhardtii* orthomolog[2]	*T.thermophila* ortholog[3]	Human ortholog[4]
1	Tubulin	α tubulin	A2E8B1	276	452	1-439	α tubulin	α tubulin	α tubulin
2	Tubulin	β tubulin	A2DC16	276	447	1-428	β tubulin	β tubulin	β tubulin
3	A-tubule	Rib72	A2GCC1	12	595	65-595	Rib72	Rib72	EFHC1/2
4	B-tubule	FAP45	A2ETR1	2	465	1-107, 108-465	FAP45	CFAP45	FAP45
5	B-tubule	FAP52	A2FVE3	6	605	1-67, 86-776	FAP52	CFAP52	FAP52
6	Inner Junction	PACRGB	A2EJQ5	6	241	18-235	PACRG	PACRG	PACRG
7	A-tubule	FAP21	A2F5C9	2	386	321-386	FAP21	FAP21	FAP21
8	Inner Junction	PACRGA	A2DAX1	6	236	42-233	PACRG	PACRG	PACRG
9	A-tubule	FAP53	A2G223	2	482	1-224, 238-470	FAP53	CFAP53	FAP53
10	A-tubule	*Tv*FAP12	A2F1C6	2	108	1-108	_	_	_
11	B-tubule	CCDC173	A2EFC9	2	455	33-355, 374-455	FAP210	CCDC173	FAP210
12	A-tubule	FAP115	A2G843	3	927	475-681	FAP115	CFAP115	_
13	A-tubule	FAP67	A2E829	2	375	1-375	FAP67	CFAP67A	NME7
14	B-tubule	*Tv*FAP35	A2DUL4	6	306	8-19, 27-306	_	_	_
15	B-tubule	*Tv*FAP40	A2DSS2	6	377	1-377	_	_	_

16	Ribbon	Rib43a	A2DEG5	2	383	1-361	Rib43a	Rib43a	RIBC2
17	B-tubule	FAP77	A2FTW1	6	283	71-283	FAP77	CFAP77	FAP77
18	A-tubule	*Tv*FAP9	A2DVD4	2	75	1-48	_	_	_
19	A-tubule	FAP127	A2FH94	2	490	1-490	FAP127	CFAP127	MNS1
20	B-tubule	Enkurin	A2E7V9	6	241	1-241	FAP106	A8I9E8	ENKUR
21	Inner Junction	FAP20	A2EAE1	12	194	1-184	FAP20	CFAP20	FAP20
22	N-DRC	DRC4*	A2FWB4	1	512	315-493	DRC4	DRC4	DRC4
23	N-DRC	DRC2*	A2GIM1	1	461	251-339, 340-411	DRC2	DRC2	DRC2
24	N-DRC	DRC1*	A2DEK4	1	633	292-370, 522-633	DRC1	DRC1	DRC1
25	External coiled coils	CFAP58*	A2FE28	1	870	480-726, 727-870	FAP189	CFAP58	FAP58
26	Radial Spoke Base	FAP253*	A2DGT8	1	396	200-270, 313-374	FAP253	CFAP253	IQUB
27	Radial Spoke Base	CCDC96*	A2DTI0	1	363	214-315, 316-361	FAP184	CCDC96	CCDC96
28	External coiled coils	CCDC39*	A2EKX3	1	998	3-37, 58-299	FAP59	CCDC39	CCDC39
29	External coiled coils	CCDC40*	A2DSS7	1	889	77-95, 105-311	FAP172	CCDC40	CCDC40
30	N-DRC/radial spoke	FAP91*	A2F6E6	1	602	305-660	FAP91	CFAP91	FAP91

Supplementary Table 3: Ligand Classification of *Tv*FAP40 Ligand. Listed are the top 10 hits identified by the cryoEM ligand classification software labeled by ligand class defined by CheckMyBlob [5].

Rank	Label	Probability
1	Rare Ligand	0.27
2	CLA-like	0.27
3	BCL	0.2
4	NAG	0.15
5	HEM or OXY + HEM	0.04
6	BLA-like	0.02
7	FUC + NAG + NAG	0.02
8	FUC + NAG	0.01
9	U5P	0.01
10	SO4-like	0.01

Supplementary Table 4: Autodock Vina Ligand Binding Hits. Listed are the top 50 hits identified by the Autodock Vina [6] library sorted by predicted free energy of binding for the TvFAP40 ligand binding pocket.

Ligand	Predicted Free Energy of Binding (kcal/mol)
Violacein	-6.3
NAD+	-5
Riboflavin	-5
CTP	-4.9
GDP	-4.9
2-(acetylamino)-1,5-anhydro-2-deoxy-3-O-beta-D-galactopyranosyl-D-arabino-Hex-1-enitol	-4.8
Alpha-Ribazole	-4.8
Guanosine	-4.8
N-Ribosylnicotinamide	-4.8
UDP	-4.8
CMP	-4.7
Dihydropteroate	-4.7
IMP	-4.7
Inosine	-4.7
N-Acetylmuramoyl-Ala	-4.7
Pseudouridine	-4.7
UDP-N-acetyl-D-glucosamine	-4.7
Uridine	-4.7
2-carboxy-3-keto-D-arabinitol-1,5-bisphosphate	-4.6
8-Oxocoformycin	-4.6
8-Oxodeoxycoformycin	-4.6
CDP-ethanolamine	-4.6
dGMP	-4.6
GTP	-4.6
IP5	-4.6
Tetrahydropteroate	-4.6
UDP-glucose	-4.6
UMP	-4.5
Adenosine	-4.5
ADP	-4.5
D-Fructose-1,6-bisphosphate	-4.5
Isopentenyladenosine	-4.5
S-Acetylphosphopantetheine	-4.5
Tetrahydrobiopterin	-4.5
Thymidine	-4.5
UDP-glucuronate	-4.5

**Urate**	-4.5
**UTP**	-4.4
**5'-Hydroxycotinine**	-4.4
**5-6-Dihydrouridine**	-4.4
**5-Acetylamino-6-formylamino-3-methyluracil**	-4.4
**Deoxyuridine**	-4.4
**Guanosine 3'-phosphate**	-4.4
**IDP**	-4.4
**N-Succinyl-L-glutamate**	-4.4
**Orotidine**	-4.4
**Sucrose**	-4.4
**Tetrahydrofolate**	-4.4
**(1-Ribosylimidazole)-4-acetate**	-4.3

Supplementary Table 5: Structural homologs of *Tv*FAP35 and *Tv*FAP40. Shown are three proteins from *Trichomonas vaginalis*, including *Tv*FAP35 and *Tv*FAP40, along with the 31 proteins found via FoldSeek [7] that matched our criteria. Many other structures with similar AlphaFold predictions can be found by searching the AlphaFold similarity clusters for each of these proteins, but only a few examples from relevant pathogens are shown here.

	**Assignment**	**UniProt ID**	**Date of Accession**	**Organism**	**Search method**
1	*Tv*FAP35	A2DUL4	March 25, 2025	*Trichomonas vaginalis*	Inspection of AlphaFold predictions for proteins in P5 fraction
2	*Tv*FAP40	A2DSS2	March 25, 2025	*Trichomonas vaginalis*	Inspection of AlphaFold predictions for proteins in P5 fraction
3	Uncharacterized	A2DG66	March 25, 2025	*Trichomonas vaginalis*	Inspection of AlphaFold predictions for proteins in P5 fraction
4	Uncharacterized	Q38A11	March 25, 2025	*Trypanosoma brucei*	FoldSeek of *Tv*FAP40 kinked coiled-coil motif
5	Uncharacterized	Q4DYR7	March 25, 2025	*Trypanosoma cruzi*	FoldSeek of *Tv*FAP40 kinked coiled-coil motif
6	Uncharacterized	G0U672	March 25, 2025	*Trypanosoma vivax* strain Y486	FoldSeek of *Tv*FAP40 kinked coiled-coil motif
7	Uncharacterized	G0U7P3	March 25, 2025	*Trypanosoma vivax* strain Y486	FoldSeek of *Tv*FAP40 kinked coiled-coil motif
8	Uncharacterized	A4IAR0	March 25, 2025	*Leishmania infantum*	FoldSeek of *Tv*FAP40 kinked coiled-coil motif
9	Uncharacterized	A0A3P3ZIB6	March 25, 2025	*Leishmania braziliensis*	FoldSeek of *Tv*FAP40 kinked coiled-coil motif
10	Uncharacterized	A0A7J6MRK3	March 25, 2025	*Perkinsus chesapeaki*	FoldSeek of *Tv*FAP40 kinked coiled-coil motif
11	Uncharacterized	A0A7J6L2D7	March 25, 2025	*Perkinsus chesapeaki*	FoldSeek of *Tv*FAP40 kinked coiled-coil motif

12	Uncharacterized	A0E9V2	March 25, 2025	*Paramecium tetraurelia*	FoldSeek of *Tv*FAP40 kinked coiled-coil motif
13	Uncharacterized	A0CNB3	March 25, 2025	*Paramecium tetraurelia*	FoldSeek of *Tv*FAP40 kinked coiled-coil motif
14	Uncharacterized	A0E9J4	March 25, 2025	*Paramecium tetraurelia*	FoldSeek of *Tv*FAP40 kinked coiled-coil motif
15	Uncharacterized	A0A0S4IWZ6	March 25, 2025	*Bodo saltans*	FoldSeek of *Tv*FAP40 kinked coiled-coil motif
16	Uncharacterized	A0A6V1QLJ6	March 25, 2025	*Heterosigma akashiwo*	FoldSeek of *Tv*FAP40 kinked coiled-coil motif
17	Uncharacterized	T0QZ48	March 25, 2025	*Saprolegnia diclina strain VS20*	FoldSeek of *Tv*FAP40 kinked coiled-coil motif
18	Uncharacterized	A0A3L6UWR8	March 25, 2025	*Aphanomyces astaci*	FoldSeek of *Tv*FAP40 kinked coiled-coil motif
19	Uncharacterized	A0A7S1BAN9	March 25, 2025	*Corethron hystrix*	FoldSeek of *Tv*FAP40 kinked coiled-coil motif
20	Uncharacterized	A0A7J7PGH9	March 25, 2025	*Scenedesmus* sp. NREL 46B-D3	FoldSeek of *Tv*FAP40 kinked coiled-coil motif
21	Uncharacterized	A0A7J7PH71	March 25, 2025	*Scenedesmus* sp. NREL 46B-D3	FoldSeek of *Tv*FAP40 kinked coiled-coil motif
22	Uncharacterized	A0A7S3QL64	March 25, 2025	*Dunaliella tertiolecta*	FoldSeek of *Tv*FAP40 kinked coiled-coil motif
23	Uncharacterized	A0A813EJG0	March 25, 2025	*Polarella glacialis*	FoldSeek of *Tv*FAP40 kinked coiled-coil motif
24	Uncharacterized	A0A812HW03	March 25, 2025	*Symbiodinium* sp. KB8	FoldSeek of *Tv*FAP40 kinked coiled-coil motif
25	Uncharacterized	A0A7S3WEM8	March 25, 2025	*Emiliania huxleyi*	FoldSeek of *Tv*FAP40 kinked coiled-coil motif
26	Uncharacterized	A0A0G4FWY3	March 25, 2025	*Vitrella brassicaformis* strain CCMP3155	FoldSeek of *Tv*FAP40 kinked coiled-coil motif
27	Uncharacterized	A0A7S4EBY1	March 25, 2025	*Pelagomonas calceolata*	FoldSeek of *Tv*FAP40 kinked coiled-coil motif
28	Uncharacterized	A0A6A0A2X9	March 25, 2025	*Haematococcus lacustris*	FoldSeek of *Tv*FAP40 kinked coiled-coil motif
29	Uncharacterized	A0A836C7C9	March 25, 2025	*Tribonema minus*	FoldSeek of *Tv*FAP40 kinked coiled-coil motif
30	Uncharacterized	A0A2K1JY97	March 25, 2025	*Physcomitrium patens*	FoldSeek of *Tv*FAP40 kinked coiled-coil motif
31	Uncharacterized	A0A6A5BUR3	March 25, 2025	*Naegleria fowleri*	FoldSeek of *Tv*FAP40 kinked coiled-coil motif
32	Uncharacterized	A0A6A5BK07	March 25, 2025	*Naegleria fowleri*	FoldSeek of *Tv*FAP40 kinked coiled-coil motif
33	Uncharacterized	A0A7J6MUA5	March 25, 2025	*Perkinsus olseni*	FoldSeek of *Tv*FAP40 kinked coiled-coil motif
34	Uncharacterized	A0A7J6LZT7	March 25, 2025	*Perkinsus olseni*	FoldSeek of *Tv*FAP40 kinked coiled-coil motif
35	Uncharacterized	A0A3P3ZIB6	March 25, 2025	*Leishmania donovani*	AlphaFold structure similarity cluster (AFDB50/MMseqs2) for *Leishmania braziliensis* protein A0A3P3ZIB6
36	Uncharacterized	A0A1E1J9F8	March 25, 2025	*Leishmania guyanensis*	AlphaFold structure similarity cluster (AFDB/FoldSeek) for *Leishmania braziliensis* protein A0A3P3ZIB6

37	Uncharacterized	E9ASV2	March 25, 2025	*Leishmania mexicana*	AlphaFold structure similarity cluster (AFDB50/MMseqs2) for *Leishmania braziliensis* protein A0A3P3ZIB6
38	Uncharacterized	A0A836FPF5	March 25, 2025	*Leishmania martiniquensis*	AlphaFold structure similarity cluster (AFDB50/MMseqs2) for *Leishmania infantum* protein A4IAR0
39	Uncharacterized	A0A836HHZ8	March 25, 2025	*Leishmania* sp. Ghana 2012 LV757	AlphaFold structure similarity cluster (AFDB50/MMseqs2) for *Leishmania infantum* protein A4IAR0
40	Uncharacterized	A0A836G1H3	March 25, 2025	*Leishmania orientalis*	AlphaFold structure similarity cluster (AFDB50/MMseqs2) for *Leishmania infantum* protein A4IAR0

Supplementary Table 6: Mass Spectrometry Information

**Data Set**	**Wildtype**
**MS reaction details**	3% Acetonitrile, 0.1% Formic Acid
**MS time course (min)**	0.25, 1, 10, 60, 480, 1440
**Control samples**	Maximally-labeled control (WT protein)
**Sample preparation**	Trypsin digestion following reduction, alkylation, acetone precipitation, detergent removal (HiPPR), desalting (C18 columns), and lyophilization.
**Injection volume**	~1.0 µg protein
**Column temperature**	4 C
**Back-exchange (mean / IQR)**	42.70% / 13.9%
**# of Peptides**	311 identified proteins, filtered to 239 cytoskeletal proteins
**Sequence coverage**	91%
**Average peptide length / Redundancy**	13.86 / 2.03
**Replicates (biological or technical)**	3 (biological)
**Repeatability**	0.046 (average standard deviation)
**Database search parameters**	TrichDB (T. vaginalis annotated proteins, version G3)
**Software used**	ProteomeDiscoverer 2.5, DeepCoil 2.0
**Statistical tests**	scipy.stats.ttest_ind (T-tests)
**Significant differences in MS (delta MS > X D)**	0.246 D (99% CI)
**Mass Spectrometry Facility**	Chem/Biochem's Molecular Instrumentation Center, UCLA
**Facility Contact**	Dr. Yu Chen (yuchenmic@g.ucla.edu)

Supplementary References

1. Jumper, J. et al. Highly accurate protein structure prediction with AlphaFold. *Nature* **596**, 583-589 (2021).
2. Ma, M. et al. Structure of the Decorated Ciliary Doublet Microtubule. *Cell* **179**, 909-922.e12 (2019).
3. Kubo, S. et al. Native doublet microtubules from Tetrahymena thermophila reveal the importance of outer junction proteins. *Nature Communications* **14**(2023).
4. Gui, M. et al. SPACA9 is a lumenal protein of human ciliary singlet and doublet microtubules. *Proceedings of the National Academy of Sciences* **119**(2022).
5. Karolczak, J. et al. Ligand identification in CryoEM and X-ray maps using deep learning. *Bioinformatics* **41**(2024).
6. Trott, O. & Olson, A.J. AutoDock Vina: Improving the speed and accuracy of docking with a new scoring function, efficient optimization, and multithreading. *Journal of Computational Chemistry* **31**, 455-461 (2010).
7. Van Kempen, M. et al. Fast and accurate protein structure search with Foldseek. *Nature Biotechnology* **42**, 243-246 (2024).
