# Supplementary material for: Structures of Native Doublet Microtubules from Trichomonas vaginalis Reveal Parasite-Specific Proteins
Source: Nat Commun. 2025 Apr 29;16:3996. doi: 10.1038/s41467-025-59369-y (PMC12041511; doi:10.1038/s41467-025-59369-y)
Supplement: Supplementary file 2 — Description of Additional Supplementary Files [file 41467_2025_59369_MOESM2_ESM.pdf]

Description of Additional Supplementary Files

File name: Supplementary Data 1
Description: Mass spectrometry Reporting of proteins in P5 sample, with radial spoke and microtubule proteins highlighted

File name: Supplementary Movie 1
Description: Overview of *Tv*-MIPs. Cross sectional view down the *Tv*-DMT with MIP and MOP densities colored. Model view of all modeled MIPs rotated to show detail and models of *Tv*FAP35 and *Tv*FAP40 in cyan and magenta respectively.

File name: Supplementary Movie 2
Description: *Tv*FAP40 ligand binding pocket. View flying into putative ligand binding site of *Tv*FAP40. Rotations around the ligand binding site with and without the cryo-EM density.
