# Supplementary material for: Structures of Native Doublet Microtubules from Trichomonas vaginalis Reveal Parasite-Specific Proteins
Source: Nat Commun. 2025 Apr 29;16:3996. doi: 10.1038/s41467-025-59369-y (PMC12041511; doi:10.1038/s41467-025-59369-y)
Supplement: Supplementary file 6 — Reporting Summary [file 41467_2025_59369_MOESM6_ESM.pdf]

nature portfolio

Corresponding author(s): Z. Hong Zhou, Patricia Johnson

Last updated by author(s): 4-2-25

Reporting Summary

Nature Portfolio wishes to improve the reproducibility of the work that we publish. This form provides structure for consistency and transparency in reporting. For further information on Nature Portfolio policies, see our Editorial Policies and the Editorial Policy Checklist.

Statistics

For all statistical analyses, confirm that the following items are present in the figure legend, table legend, main text, or Methods section.

n/a	Confirmed	
x	☐	The exact sample size (n) for each experimental group/condition, given as a discrete number and unit of measurement
x	☐	A statement on whether measurements were taken from distinct samples or whether the same sample was measured repeatedly
x	☐	The statistical test(s) used AND whether they are one- or two-sided<br>*Only common tests should be described solely by name; describe more complex techniques in the Methods section.*
x	☐	A description of all covariates tested
x	☐	A description of any assumptions or corrections, such as tests of normality and adjustment for multiple comparisons
x	☐	A full description of the statistical parameters including central tendency (e.g. means) or other basic estimates (e.g. regression coefficient) AND variation (e.g. standard deviation) or associated estimates of uncertainty (e.g. confidence intervals)
x	☐	For null hypothesis testing, the test statistic (e.g. F, t, r) with confidence intervals, effect sizes, degrees of freedom and P value noted<br>*Give P values as exact values whenever suitable.*
x	☐	For Bayesian analysis, information on the choice of priors and Markov chain Monte Carlo settings
x	☐	For hierarchical and complex designs, identification of the appropriate level for tests and full reporting of outcomes
x	☐	Estimates of effect sizes (e.g. Cohen's d, Pearson's r), indicating how they were calculated

Our web collection on statistics for biologists contains articles on many of the points above.

Software and code

Policy information about availability of computer code

Data collection	SerialEM
Data analysis	Proteome Discoverer 2.5, CryoSPARC v3.3.1, RELION 4.0, Topaz v0.2.5 (integrated with RELION), Phenix 1.21.2, UCSF ChimeraX 1.6-1.8, ISOLDE 1.6.0, Coot 0.9.8.8, FoldSeek v9, Clustal Omega v1.2.4, PUResNet 2.0, LigandRecognizer, AutoDock Vina v1.2.0, AlphaFold2, DeepTracer, Protein BLAST, ModelAngelo v1.0.12, cryoID v1.0, OpenBabel 3.1.0, ESPript 3.0, DeepCoil 2.0

For manuscripts utilizing custom algorithms or software that are central to the research but not yet described in published literature, software must be made available to editors and reviewers. We strongly encourage code deposition in a community repository (e.g. GitHub). See the Nature Portfolio guidelines for submitting code & software for further information.

Data

Policy information about availability of data

All manuscripts must include a data availability statement. This statement should provide the following information, where applicable:

- Accession codes, unique identifiers, or web links for publicly available datasets
- A description of any restrictions on data availability
- For clinical datasets or third party data, please ensure that the statement adheres to our policy

The cryoEM map data generated in this study have been deposited in the Electron Microscopy Data Bank (EMDB) under the following accession codes: the 16, 48, and 96 nm repeats under EMD-46642 (https://www.ebi.ac.uk/emdb/EMD-46642), EMD-46643 (https://www.ebi.ac.uk/emdb/EMD-46643), and EMD-46636 (https://www.ebi.ac.uk/emdb/EMD-46636), respectively. The composite cryoEM map of the 48 nm repeat is available under EMD-46580 (https://www.ebi.ac.uk/

emdb/EMD-46580), and the coordinates for the complete atomic models are available under PDB accession code 9D5N (https://www.rcsb.org/structure/unreleased/9D5N). Focused cryoEM maps for the 8 nm TvOJMOP1, 16 nm TvFAP40, and 96 nm N-DRC refinements are available at the EMDB under accession numbers EMD-46634 (https://www.ebi.ac.uk/emdb/EMD-46642), EMD-46633 (https://www.ebi.ac.uk/emdb/EMD-46633), and EMD-46635(https://www.ebi.ac.uk/emdb/EMD-46635), respectively. Previously published 48nm repeat doublet structures from Clamydomonas, 6u42, (https://www.rcsb.org/structure/6U42), Bovine, 8otz (https://www.rcsb.org/structure/8OTZ), and Tetrahymena, 8g2z https://www.rcsb.org/structure/8G2Z), were used as reference models for comparison in our study. Reconstruction of the TvRS head and neck is available under EMDB accession number EMD-48446 (https://www.ebi.ac.uk/emdb/EMD-48446). The mass spectrometry data used in this study have been deposited in the MassIVE database under accession code MSV000096489, ftp://MSV000096747@massive.ucsd.edu with the username: MSV000096747_reviewer and the password is 7Zo20S9boDgXG82x.

Research involving human participants, their data, or biological material

Policy information about studies with human participants or human data. See also policy information about sex, gender (identity/presentation), and sexual orientation and race, ethnicity and racism.

Reporting on sex and gender	Not applicable for this study, no human research was conducted
Reporting on race, ethnicity, or other socially relevant groupings	Not applicable for this study, no human research was conducted
Population characteristics	Not applicable for this study, no human research was conducted
Recruitment	Not applicable for this study, no human research was conducted
Ethics oversight	Not applicable for this study, no human research was conducted

Note that full information on the approval of the study protocol must also be provided in the manuscript.

Field-specific reporting

Please select the one below that is the best fit for your research. If you are not sure, read the appropriate sections before making your selection.

☒ Life sciences ☐ Behavioural & social sciences ☐ Ecological, evolutionary & environmental sciences

For a reference copy of the document with all sections, see nature.com/documents/nr-reporting-summary-flat.pdf

Life sciences study design

All studies must disclose on these points even when the disclosure is negative.

Sample size	No statistical methods related to sample size calculation were relevant to our study. For mass spectrometry, three independent biological replicates were prepared from separately cultured Trichomonas vaginalis G3 stocks under identical media and growth conditions. CryoEM reconstructions were generated from 30,834 dose-fractionated movies collected on a Titan Krios microscope equipped with a K3 detector and energy filter. Fresh buffers and reagents were prepared for each replicate to ensure sample consistency.
Data exclusions	Individual reconstructions were formed from the appropriate subsets of particles obtained via classification. Particles that were classified into poor quality classes were excluded. All of these choices were performed according to standard cryoEM practices.
Replication	The conserved features of our cryoEM maps are consistent with previously published homologous structures, which were determined independently. For many individual reconstructions, multiple refinements led to effectively identical results. Many proteins were able to be accurately atomically modeled to a specific amino acid sequence, which is independent of the cryoEM data processing workflow. For mass spectrometry experiments, three independent biological replicates were prepared from separately cultured T. vaginalis G3 stocks, and only proteins consistently identified across all three replicates were included in further analyses. For ligand docking analysis, multiple ligands and structural variants were screened independently across repeated docking runs, including metabolite library screening. Consistent binding affinities and docking poses across runs confirmed the validity of our results
Randomization	Manual randomization was not applicable for this study. Splitting of data to calculate gold standard Fourier Shell Correlation necessarily included data randomization and independent refinement.
Blinding	Not applicable for this study.

Reporting for specific materials, systems and methods

We require information from authors about some types of materials, experimental systems and methods used in many studies. Here, indicate whether each material, system or method listed is relevant to your study. If you are not sure if a list item applies to your research, read the appropriate section before selecting a response.

Materials & experimental systems

n/a	Involved in the study
☒	☐ Antibodies
☐	☒ Eukaryotic cell lines
☒	☐ Palaeontology and archaeology
☒	☐ Animals and other organisms
☒	☐ Clinical data
☒	☐ Dual use research of concern
☒	☐ Plants

Methods

n/a	Involved in the study
☒	☐ ChIP-seq
☒	☐ Flow cytometry
☒	☐ MRI-based neuroimaging

Eukaryotic cell lines

Policy information about cell lines and Sex and Gender in Research

Cell line source(s)	Trichomonas vaginalis strain G3 was purchased from ATCC, # PRA98.
Authentication	Not applicable, as T. vaginalis G3 is a well-established laboratory strain.
Mycoplasma contamination	Not applicable, as T. vaginalis is a protozoan parasite, not a mammalian cell line.
Commonly misidentified lines (See ICLAC register)	None were used in this study.

Plants

Seed stocks	Not applicable for this study
Novel plant genotypes	Not applicable for this study
Authentication	Not applicable for this study
